# Antigen processing and presentation: Evolution from a bird's eye view^☆^

**DOI:** 10.1016/j.molimm.2012.10.030

**Published:** 2013-09

**Authors:** Jim Kaufman

**Affiliations:** a Department of Pathology, University of Cambridge, Tennis Court Road, Cambridge CB2 1QP, UK; b Department of Veterinary Medicine, University of Cambridge, Madingley Road, Cambridge CB3 0ES, UK

**Keywords:** Antigen processing, Antigen presentation, Major histocompatibility complex, MHC, Class I, Transporters associated with antigen presentation, TAP, Tapasin, Bird, Chicken, Non-mammalian, Vertebrate, Evolution, Adaptive immunity

## Abstract

Most detailed knowledge of the MHC outside of mammals has come from studies of chickens, originally due to the economic importance of the poultry industry. We have used our discoveries about the chicken MHC to develop a framework for understanding the evolution of the MHC, based on the importance of genomic organisation for gene co-evolution. In humans, MHC class I molecules are polymorphic and determine the specificity of peptide presentation, while the molecules involved in antigen processing are functionally monomorphic. The genes for tapasin, transporters associated with antigen presentation (TAPs) and inducible proteasome components (LMPs) are located in and beyond the class II region, far away from the class I genes in the class I region. In contrast, chickens express only one class I locus at high levels, which can result in strong MHC associations with resistance to particular infectious pathogens. The chicken TAP and tapasin genes are located very close to the class I genes, and have high levels of allelic polymorphism and moderate sequence diversity, co-evolving their specificities to work optimally with the dominantly expressed class I molecule. The salient features of the chicken MHC are found in many if not most non-mammalian species examined, and are likely to represent the ancestral organisation of the MHC. Comparison with the MHC organisation of humans and typical mammals suggests that a large inversion brought the class III region into the middle of the MHC, separating the antigen processing genes from the class I gene, breaking the co-evolutionary relationships and allowing a multigene family of well-expressed class I genes. Such co-evolution in the primordial MHC was likely responsible for the appearance of the antigen presentation pathways and receptor–ligand interactions at the birth of the adaptive immune system. Of course, much further work is required to understand this evolutionary framework in more detail.

## Highlights

► Single MHC molecules in chicken versus multiple MHC molecules in mammals. ► Single MHC molecules in chicken strongly determine immune response. ► Single chicken class I due to co-evolution with polymorphic antigen processing genes. ► Co-evolution in chicken MHC due to different genomic organisation. ► Chicken MHC like ancestral MHC, with inversion in lineage to mammals.

## 1 Introduction

After a century of careful investigation into the genetics, genomics, biochemistry, cell biology and cellular immunology of the adaptive immune response, there is a clear picture of antigen processing and the subsequent antigen presentation by major histocompatibility complex (MHC) class I and class II molecules to T lymphocytes and natural killer (NK) cells. However, almost all of this work has focused on a few mammals important for biomedicine: humans, mice and to a lesser extent rats. To what extent are the pictures found in textbooks true for the other animals?

The MHC, MHC class I and class II molecules, and some of the antigen processing genes are found in most jawed vertebrates, from sharks to humans. However, our knowledge of antigen processing and presentation outside of mammals is almost exclusively due to examination of one non-mammalian vertebrate: the chicken (Kaufman, 2008). In fact, many early discoveries in immunology, virology and cancer were made using chickens, at least in part due to their economic importance. Among those early discoveries was the chicken MHC, originally described as the B blood group, before the identification of the human MHC. An enormous literature over 60 years describing pathogens, host genetics and immunological phenomenology has meant that the chicken is by far the best studied non-mammalian vertebrate in terms of disease resistance and vaccine response (although with the rise of aquaculture, fish are catching up fast).

## 2 Current status

Building on the work of many other colleagues, we have used our discoveries about the chicken MHC (a region called the BF–BL region) to develop a framework to understand some of the important features in the evolution of the MHC, from the earliest beginnings to typical mammals. In Table 1, some well-known properties of the MHC of humans and most other mammals are listed on the left, with the contrasting features that we first discovered in chickens on the right (Kaufman, 2008; Kaufman et al., 1995, 1999).

Starting at the bottom of Table 1, the human MHC is known as the region with the most associations with disease, many of which can be explained by the polymorphism in class I and class II genes. However, most of these associations (and the strongest) are with autoimmune disease, and the associations with resistance to infectious disease are much fewer and weaker. It is not to say that important associations of the human MHC do not exist, but it has taken the efforts of truly gifted researchers to demonstrate them satisfactorily. In contrast, already 60 years ago, avian health researchers were stumbling on many strong associations with the B blood group.

One explanation for these strong associations of the chicken MHC with disease resistance and vaccine response might be the fact that, despite having multiple MHC genes, only one classical class I gene and one classical class II gene are expressed at a high level. Indeed, the BF–BL region represents a very small and compact MHC, and other genes with important roles in disease resistance by the human MHC (such as TNF, C2, and factor B) are found elsewhere in the chicken genome, or thus far not at all. In this view, the multigene families of well-expressed class I and class II genes, along with the other disease resistance genes, lead to most human MHC haplotypes conferring more-or-less resistance to most pathogens, which reads out as weak genetic associations. In contrast, an individual chicken might live or die based on the properties of their single dominantly expressed class I and class II alleles, which reads out as strong genetic associations.

This view leads immediately to the question of why the chicken does not express all of the classical class I and class II genes at a high level, so that it enjoys the same protection as humans and other mammals. We believe that this is deeply rooted in the mechanisms of antigen processing and peptide loading, some features of which differ markedly between humans and chickens (Table 1, middle section). For the human class I pathway, the transporters associated with antigen presentation (TAP), tapasin and inducible proteasome component (LMP) genes are all nearly monomorphic, with no obvious functional differences between alleles. This means that the antigen processing and peptide loading machinery has evolved to work with many alleles of the multigene family of human class I molecules. In chickens, the LMP genes have not been found, but the TAP and tapasin genes all have high allelic polymorphism and moderate sequence diversity. Thus far, we have found that each chicken MHC haplotype has particular alleles of TAP (and most likely tapasin) genes which work optimally with the single dominantly expressed class I molecule of that haplotype. In other words, chickens have stable haplotypes of polymorphic interacting genes which have co-evolved to work together, compared to humans in which the antigen processing and peptide loading genes have evolved as “average best fits” for all classical class I molecules.

The basis for this co-evolution appears to be the rarity of recombination between TAP, tapasin and class I genes, which is due at least in part to the organisation of the chicken MHC (Table 1, middle and top sections). The human MHC is a large and complex region, with the TAP and LMP genes in the class II region and the tapasin gene in the extended class II region, both separated by the class III region from the class I genes located in the class I region. As a result, the rate of recombination across the human MHC is estimated at 2–4% (that is, the recombinational distance is 2–4 cM). In contrast, the chicken MHC is relatively simple and compact, with the class III region outside of the class I region and class II regions, and with the TAP genes flanked by the class I genes in a tiny class I region. Moreover, the level of recombination across the BF–BL region is extremely low, although recent work suggests that so-called gene conversion (in the sense currently in vogue by the genomics field) does happen at some, thus far unknown, rate.

It was the simplicity and compact nature of the chicken MHC that allowed these important features to be easily discerned, but they are found in many if not most non-mammalian vertebrates, albeit at differing levels of proof (Kaufman, 1999, 2011). For instance, the duck has polymorphic TAP genes right next to five class I genes, only one of which is expressed at a high level. In frogs (at least Xenopus genus), the MHC is organised as we first described in the chicken, with at least two allelic lineages of TAP genes closely linked to a single class I gene. In the zebrafish, the classical class I, TAP, tapasin and LMP genes are all closely linked together in a different locus than the class II and class III genes, and in the Atlantic salmon, there is but a single classical class I gene which is strongly associated with resistance to viral but not bacterial disease. In sharks, many MHC haplotypes have a single classical class I gene. Even in one marsupial, the American opossum, the organisation of the MHC mirrors what was described for chickens.

It seems most likely that the salient features we originally described for the chicken MHC reflect the ancestral organisation of the MHC, and it was placental mammals which changed. An obvious scenario is that the typical non-mammalian organisation of class II–class I–class III region was re-arranged by an inversion in which the class III region swung into the middle of the class I region, with the class I gene(s) swung to the outside, but with the TAP, tapasin and LMP genes left behind in what eventually became part of the class II region. At this point, there would have been a strong selection for TAP, tapasin and LMP genes that could accommodate any class I allele, and eventually a multigene class I family. Superimposed on this broad history would be many secondary evolutionary events, some of which affected these two strategies of MHC organisation. For instance, the TAP and classical class I genes in the rat MHC are located closely enough to allow a limited level of co-evolution, which almost certainly arose by a secondary evolutionary event: a rodent-specific translocation of classical class I genes (giving rise to the mouse H-2K region and rat RT1-A region, with a maintenance of classical class I regions in the mouse D region but silencing in the rat equivalent). Conversely, it appears that another marsupial, the Tammar wallaby, has undergone secondary evolutionary events to separate class I genes from TAP genes as in typical placental mammals, but in this case the TAP genes remain in the MHC and the classical class I genes have fled to the telomeres of other chromosomes (Siddle et al., 2009). Another completely different and poorly understood strategy for the MHC is the presence of many tens of classical class I genes all expressed at a low level, with class II gene polymorphism reduced or the genes absent altogether (as in a salamander, the Mexican axolotl, and a group of teleost fish including the Atlantic cod, see Kaufman, 1999, 2011; Star et al., 2011).

A final point is that the TAP, tapasin, inducible proteasome component and class I genes were likely present in the ancestral MHC in order to set up the antigen processing and presentation pathway in the first place. In this view, there was a time in which the ancestors of these genes did not have their current functions, and needed to co-evolve in order to begin to work together, with genetic linkage being the best way in which to preserve combinations of genes which worked optimally together. A very similar idea was originally suggested for setting up metabolic pathways in the proposed RNA world. Moreover, the presence of at least one lectin-like NK cell receptor gene in the chicken MHC (Kaufman et al., 1999) suggests that not only ligands but receptors were originally present in the ancestral MHC in order to co-evolve as a functional pathway. Therefore, we would expect that T cell receptor and antibody genes were originally present in the ancestral MHC, and these and many other important genes were redistributed by the two rounds of genome-wide duplication at the base of the vertebrates, silenced in different genetic loci for each genetic lineage of animals, as well as suffering other re-arrangements due to recombination as secondary events. In this view, the primordial MHC was the birthplace of the adaptive immune system, and has been breaking apart ever since (Kaufman et al., 1995; Kaufman, 2011).

## 3 Future perspectives

The discoveries based on the chicken MHC summarised above have led to a plausible framework to explain the evolution of the MHC from an ancestral state, with an emphasis on the role of co-evolution of genetically linked genes both in developing the pathways of antigen processing and presentation and in maintaining the haplotypes of polymorphic interacting genes found in non-mammalian vertebrates. However, it is just a framework, and much remains to be done at every step outlined in Table 1.

Starting at the bottom of the table, this framework rests on the observation that there is a single dominantly expressed class I molecule expressed by all chicken MHC haplotypes, which leads to lack of response to particular (strains of) pathogens by particular chickens. However, chickens have an “expression level polymorphism”, in which the level of class I molecules on the surface of cells varies between haplotypes, with the lowest expressing haplotypes being most resistant to the oncogenic Marek's disease virus. We have found the dominantly expressed class I molecules of such low expressing haplotypes remodel their binding sites, so that many more peptides with different sequences can be presented (Kaufman et al., 1995; Koch et al., 2007). Is this an adaptation so that one class I molecule can act like many, and if so, what is the advantage of having class I molecules with very fastidious peptide motifs as are found in the high expressing haplotypes?

In terms of real biology, the most important implication from this framework is that the genomic structure of an MHC can lead to a particular strategy of resistance to disease. In particular it purports to explain the oft-repeated notion that the human MHC has strong associations with autoimmune disease while the chicken MHC has strong associations with infectious disease. How secure is this distinction—could it be based on sampling error and apophenia? In particular, the most detailed studies of humans are in Europe and North America, where ageing populations and rocketing rates of autoimmunity may give a different picture than chickens living close together in a pathogen-infested barn. Can these questions be framed clearly and investigated in a quantitatively convincing way?

In the middle of the table, the presence of a single dominantly expressed class I molecule in the chicken is explained by co-evolution of polymorphic interacting genes (and at the top of the table, this same important concept is used to explain the initial set-up of the whole system of antigen processing and presentation). But how does it work at the level of the interacting gene products? We have been able to relate the peptide translocation specificities and the sequences of the TAP alleles (Walker et al., 2011) by a model of the TAP heterodimer based on ABC transporters, and this may give us insight into the mechanisms that underlie the specificity of human TAPs. A similar picture may emerge from chicken tapasin alleles, although a true picture presumably requires reconstruction of a peptide-loading complex, for instance by using insect cells.

In the top section of the table, genomic organisation is used to explain the kind of co-evolution found in the chicken MHC versus the human MHC. But how does it work in practice? In particular, a change in one member of an interacting gene pair presumably requires a compensatory change in the other member in order to maintain optimal function. Thus the rate of evolutionary change should be slower in the chicken MHC than in the human MHC. Can this be measured? How in fact do new chicken MHC haplotypes arise? What features of the MHC were required for the first placental mammal to recover from the proposed inversion of the MHC? Can more general and tractable systems be set up to measure the quantitative relationship of recombination and co-evolution?

All of these ideas are based on work with the chicken class I system, but chickens also have a single dominantly expressed class II molecule. Do the same concepts involving co-evolution explain this phenomenon?

And finally, at the top of the table, the generality of this model is based primarily on work with the chicken, supported by examples scattered through the non-mammalian vertebrates, each at a very incomplete level of analysis compared to the chicken. No other non-mammalian vertebrate has been examined at this level of detail from genes, genetics and genomics, to biochemistry and cell biology, to cellular immunology and population genetics involving natural pathogens infecting a natural host. Clearly, the picture will benefit from detailed careful work in a variety of other non-mammalian animals.

## Figures and Tables

**Table 1:** At least two alternative genomic organisations which lead to differing functions of the MHC.

Humans (and most placental mammals)	Chickens (and most non-mammalian vertebrates)
MHC class III separates I and II regions: large, complex, with many genes, pseudogenes, and repetitive elements	MHC arranged differently: class II region outside of class II and class I regions, TAPs close to class I
Relatively frequent recombination, leading to polymorphic class I and II, but interacting genes monomorphic (average best fit)	Very little recombination, leading to stable haplotypes of polymorphic interacting genes (co-evolution)
Multigene families of class I and II, giving strong associations with autoimmunity (but weak associations with infectious pathogens)	Dominantly expressed class I and II, giving strong associations with infectious pathogens
